# Refractory Ventricular Fibrillation in the Context of ST-Elevation Myocardial Infarction (STEMI): A Case Report of Survival With Double Sequential External Defibrillation and Percutaneous Coronary Intervention

**DOI:** 10.7759/cureus.102342

**Published:** 2026-01-26

**Authors:** Chuang Lin Wang Kong, Jose Gerardo Lopez Saenz, Daniel Casares Fallas

**Affiliations:** 1 Medicine and Surgery, University of Costa Rica, San Jose, CRI; 2 Anesthesiology, Hospital Mexico, San Jose, CRI; 3 Intensive Care Unit, Hospital Rafael Angel Calderon Guardia, San Jose, CRI

**Keywords:** advanced cardiac life support, defibrillators, myocardial infarction, percutaneous coronary intervention, ventricular fibrillation

## Abstract

Refractory ventricular fibrillation (RVF) is a life-threatening arrhythmia that remains a challenge despite adherence to advanced resuscitation protocols. We present the case of a previously healthy 42-year-old man who experienced in-hospital cardiac arrest due to RVF following an acute inferolateral myocardial infarction. After multiple unsuccessful conventional defibrillation attempts, double sequential external defibrillation (DSED) with lateral decubitus position was employed as a salvage maneuver, resulting in return of spontaneous circulation (ROSC). The patient subsequently underwent successful thrombolysis and coronary revascularization with complete neurological and cardiac recovery. This case exemplifies the potential utility of DSED in treating RVF and emphasizes the importance of timely recognition, intervention, and post-resuscitation care in improving outcomes.

## Introduction

Despite advancements in medical technology, cardiorespiratory arrest still carries a high mortality rate, especially when it occurs outside the hospital setting [1]. Ventricular fibrillation (VF) is among the most common and lethal arrhythmias encountered in such scenarios [1]. When VF persists despite three or more defibrillation attempts, it is classified as refractory VF (RVF). Although relatively rare - accounting for approximately 4% of VF cases - RVF is associated with a mortality rate as high as 97% [2,3].

Double sequential external defibrillation (DSED) has been described as a potential therapeutic alternative in these critical cases [1]. This technique involves the use of two defibrillators to deliver rapid sequential shocks, with one electrode pad set placed in the standard anterolateral position and a second set in the anteroposterior position [4,5].

This report describes the case of an atypical presentation of an inferolateral infarction in a young male patient with an unknown medical history, who experienced in-hospital cardiac arrest due to RVF that did not respond to multiple attempts at conventional defibrillation. Return of spontaneous circulation (ROSC) was successfully achieved following the application of DSED with two sets of electrode paddles. Given the limited representation of this technique in current resuscitation guidelines, this case highlights the potential life-saving value of DSED and underscores the importance of further exploration and documentation of its clinical use.

## Case presentation

A 42-year-old male patient, a service supervisor at a peripheral hospital with no significant past medical history, collapsed in a hospital hallway. The event was witnessed by a physician, and the patient was immediately transferred to the resuscitation room. In the days preceding the event, the patient had reported chest pain, dyspnea, general malaise, and a subconjunctival hemorrhage.

Upon initial evaluation in the resuscitation room, the patient was pulseless, exhibited marked cyanosis, and had miotic pupils. High-quality chest compressions were promptly initiated. The patient was intubated with fentanyl, midazolam, and atracurium and placed on mechanical ventilation. After the first cycle of cardiopulmonary resuscitation (CPR), he displayed agonal respirations and a sinus bradycardic rhythm on the monitor. However, he subsequently relapsed into cardiac arrest, presenting with pulseless VF.

**Figure 1:**
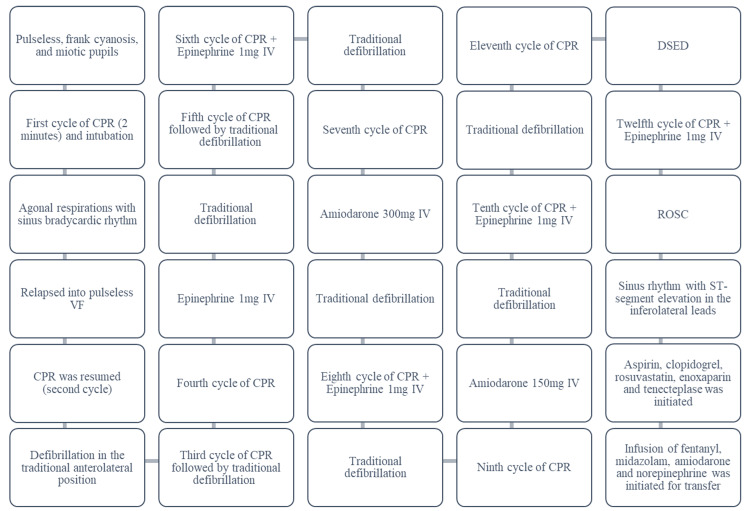
Timeline of interventions and clinical events in the initial resuscitation room ROSC: return of spontaneous circulation; CPR: cardiopulmonary resuscitation; VF: ventricular fibrillation; DSED: double sequential external defibrillation; IV: intravenous

CPR was resumed. Initial defibrillation attempts were made using a single set of paddle electrodes delivering 200 J of energy in the standard anterolateral position, with the patient in the supine position, using a manual defibrillator (Nihon Kohden SB-831V, Nihon Kohden Corporation, Tokyo, Japan), as the hospital lacked a defibrillator with adhesive pads. Repeated doses of intravenous (IV) epinephrine 1 mg were administered, and IV amiodarone was given (300 mg during the ninth CPR cycle, followed by 150 mg after the tenth cycle). Despite these efforts, VF persisted for 20 minutes (Figure 1).

**Figure 2:**
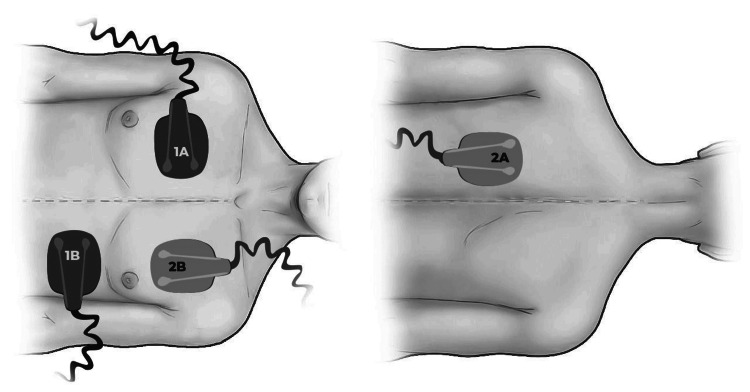
Electrical paddle positioning in double sequential external defibrillation One set of paddles was placed in the traditional anterolateral position (1A and 1B), and a second set was placed in the anteroposterior position (2A and 2B). Source: Authors' own elaboration

As a final measure, DSED was attempted. The patient was repositioned to lateral decubitus, and a second clinician applied an additional set of electrical paddles in the anteroposterior position (Figure 2), while the first clinician maintained the original anterolateral paddles, both with 200 J of energy. Following this maneuver and an additional cycle of CPR, the patient achieved ROSC.

Post-resuscitation laboratory testing revealed hyperglycemia, mild leukocytosis, and severe metabolic acidosis with elevated serum lactate levels (Table 1). The electrocardiogram (ECG) demonstrated sinus rhythm with ST-segment elevation in the inferolateral leads, and the mean arterial pressure was 60 mmHg. Due to a strong suspicion of ST-elevation myocardial infarction (STEMI), aspirin 300 mg, clopidogrel 300 mg, rosuvastatin 40 mg, and enoxaparin 40 mg were administered, and thrombolysis was performed with tenecteplase 50 mg. The patient was transferred to a tertiary center for urgent percutaneous coronary intervention (PCI) while sedated with fentanyl (0.1 mg/h) and midazolam (10 mg/h) and supported with infusions of amiodarone (1 mg/min) and norepinephrine (0.1 μg/kg/min).

**Table 1:** Results of relevant laboratory tests before and after arrival at the referral center, including the respective reference ranges PCO_2_: partial pressure of carbon dioxide; PO_2_: partial pressure of oxygen; NT-proBNP: N-terminal pro B-type natriuretic peptide

Laboratory Test	Initial Test	Post-Resuscitation Test	Reference Range (Units)
Hemoglobin	17.0	16.0	12.5-15.0 (g/dL)
Leukocyte count	14,010	32,360	5,000-10,000 (μL)
Platelet count	245,000	391,000	150,000-450,000 (μL)
Glucose	210.0	164.0	70.0-100.0 (mg/dL)
Creatinine	1.11	1.27	0.60-1.20 (mg/dL)
Urea nitrogen	12.2	18.0	7.0-25.0 (mg/dL)
pH	6.96	7.32	7.35-7.45
PCO_2_	44.0	37.0	32.0-48.0 (mmHg)
PO_2_	363.0	97.0	83.0-108.0 (mmHg)
Bicarbonate	9.9	19.1	21.0-28.0 (mmol/L)
Lactate	15.0	3.4	1.0-1.4 (mmol/L)
C-reactive protein	<0.25	<0.25	<0.50 (mg/dL)
Troponin I	5.0	31,603.0	<17.5 (pg/mL)
NT-proBNP	-	57	<125 (pg/mL)

**Figure 3:**
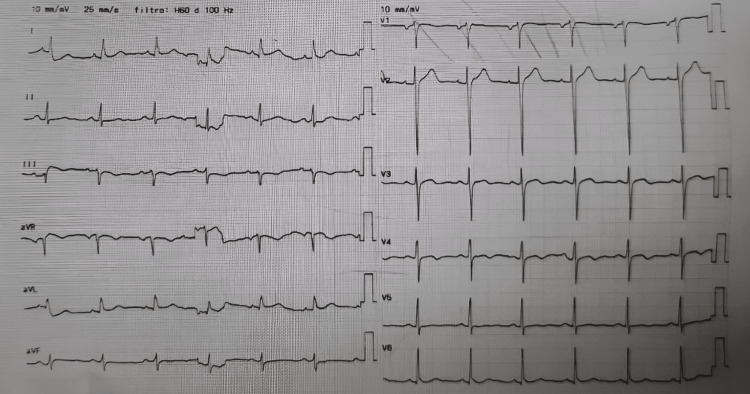
Electrocardiogram upon arrival at the referral center Sinus rhythm without ischemic or repolarization changes.

Upon arrival at the referral center, the patient’s hemodynamics had stabilized (blood pressure 115/76 mmHg), with no signs of distal hypoperfusion. The ECG showed sinus rhythm without ischemic or repolarization abnormalities (Figure 3). Laboratory studies revealed markedly elevated troponin I levels (31,603 ng/L) with normal N-terminal pro B-type natriuretic peptide (NT-proBNP) levels. The patient was taken to the catheterization laboratory, where PCI identified a severe 90% occlusion in the mid-segment of the left anterior descending artery (at the bifurcation with the diagonal branch), extending to the distal segment, requiring placement of two stents (Orsiro Mission, BIOTRONIK AG, Bülach, Switzerland; XIENCE Sierra, Abbott Vascular Inc., Tipperary, Ireland). The dominant right coronary artery demonstrated an 80% occlusion at the origin of the posterior interventricular (posterior descending) artery, requiring placement of one stent (Orsiro Mission, BIOTRONIK AG, Bülach, Switzerland). The circumflex artery showed no significant lesions. Thrombolysis in Myocardial Infarction (TIMI) grade 3 flow was restored in all treated vessels, indicating normal epicardial perfusion, and the procedure was completed without immediate complications.

Post-procedure echocardiography revealed a left ventricular ejection fraction (LVEF) of 58%, normal ventricular dimensions, mild biatrial dilation, and no pericardial effusion. The patient was extubated 48 hours later and discharged home 10 days after the event.

At one-month cardiology follow-up, the patient remained asymptomatic, with no recurrent chest pain, dyspnea, or syncope. An ECG showed sinus rhythm with residual T-wave inversions in leads II, III, and augmented voltage foot (aVF), consistent with prior inferior ischemia. Transthoracic echocardiography demonstrated preserved LVEF (55%) without regional wall motion abnormalities and mild dilation of both atria. A stress test demonstrated good functional capacity (12.9 metabolic equivalents (METs)), achieving 85% of the predicted maximum heart rate without symptoms or ischemic changes.

## Discussion

This case documents an unusual and critical event in a young male patient with no known cardiovascular history who presented with an inferolateral myocardial infarction complicated by sustained VF, successfully resuscitated using DSED, followed by timely thrombolysis and definitive revascularization. The patient achieved full neurological and cardiac recovery, with no significant short-term sequelae, demonstrating a favorable outcome in an otherwise highly fatal clinical scenario.

Acute myocardial infarction (AMI) remains a diagnostic challenge in young patients. Although rare, it carries high mortality, especially when associated with malignant arrhythmias such as RVF [3].

In this context, DSED - with the application of two nearly simultaneous shocks from two defibrillators using anterolateral and anteroposterior thoracic vectors - has emerged as a rescue strategy for pulseless RVF unresponsive to the standard advanced CPR algorithm [4]. Although the evidence remains limited and DSED is not currently part of standard resuscitation guidelines, the last five years have seen an increase in reported successful cases of RVF with complete neurological recovery using DSED - mostly isolated case reports from various countries - reflecting growing global interest in its future incorporation into guidelines. These reports generally involve young patients who suffer sudden cardiac arrest with RVF that fails to respond to the standard advanced cardiac life support (ACLS) algorithm. Consequently, DSED was used as an alternative measure, successfully reversing the malignant arrhythmia, and all those patients achieved good clinical outcomes [5-8].

Most previous DSED studies describe the use of adhesive electrode pads with the patient in the supine position. In this case, due to limited resources, the procedure was performed using two pairs of electrode paddles with the patient in lateral decubitus, yet the intervention remained effective [4-9].

Recently, randomized clinical evidence - most notably the Canadian DOSE-VF (Double Sequential External Defibrillation for Refractory Ventricular Fibrillation) trial published in The New England Journal of Medicine - demonstrated higher arrhythmia-termination rates, increased survival to hospital discharge, higher rates of ROSC, and more favorable neurological outcomes in patients with RVF treated with DSED compared to conventional defibrillation [4]. In addition, ongoing randomized controlled trials - including DOUBLE-D (Early Double Sequential Defibrillation in Out-of-Hospital Cardiac Arrest) and STRAT-DEFI (Strategies for Defibrillation During Out-of-Hospital Cardiac Arrest) - are further investigating the impact of DSED in RVF and other initial arrest rhythms, with some trials proposing DSED as a potential first-line shock strategy in out-of-hospital cardiac arrest (DUALDEFIB (Initial Double Sequential External Defibrillation in Out-of-Hospital Cardiac Arrest)). These studies are expected to provide greater clarity regarding the indications, timing, and clinical value of DSED in cardiac arrest management [9].

This case also highlights the importance of immediate event recognition, high-quality chest compressions, and the availability of trained personnel in the hospital setting-factors that should never be overlooked [10,11]. Furthermore, the use of antiplatelet and fibrinolytic therapy, along with early transfer to the catheterization laboratory for successful PCI, provided definitive etiological treatment of the event [12].

Therefore, this case emphasizes several key points for emergency department professionals: first, adherence to conventional resuscitation guidelines with trained teams, advanced equipment, appropriate defibrillation strategies, prompt epinephrine administration, and investigation of possible underlying causes [11]; second, AMI should always be considered a potential cause of malignant arrhythmias, regardless of the patient’s age, sex, or risk factors [12]; and third, DSED should be regarded as a potentially game-changing alternative for RVF [4,9]. Moreover, early coronary reperfusion following ROSC remains essential in AMI for optimal recovery [11,12].

Beyond its clinical implications, this report underscores the need for further research to better define the role of DSED within advanced cardiac life support, including standardized patient-selection criteria, optimal timing, and safety profiles, to guide future recommendations.

One limitation of this case is the lack of genetic testing or advanced cardiac imaging to rule out conditions that predispose young patients to arrhythmias, such as arrhythmogenic right ventricular dysplasia or channelopathies. Nevertheless, the angiographic finding of an obstructive coronary lesion appears sufficient to explain the clinical presentation in this patient.

## Conclusions

This case illustrates the potential role of DSED as an adjunctive intervention in selected instances of RVF, particularly in in-hospital settings where appropriate equipment and trained personnel are readily available. In this patient, the temporally associated ROSC following DSED - delivered in combination with prompt pharmacologic and mechanical reperfusion strategies - highlights how coordinated, time-sensitive interventions may facilitate recovery even after prolonged cardiac arrest in high-risk scenarios. Although the outcome in this case is encouraging, it should be interpreted with caution, as it represents a single clinical observation rather than evidence of generalizable effectiveness.

The case further underscores the importance of maintaining a high index of suspicion for acute coronary syndromes in younger adults who present with unexplained cardiovascular collapse and reinforces that early, intensive management - including thrombolysis when indicated and timely catheterization - may contribute to favorable neurological and cardiac outcomes when effectively coordinated. Finally, this report emphasizes the need for further systematic investigation of DSED across diverse clinical settings to better delineate patient selection criteria, optimal timing of intervention, safety considerations, and the potential integration of DSED into future resuscitation guidelines.
